# Establishing an Individual Writing Practice

**Published:** 2017-05-01

**Authors:** Lydia T. Madsen, Kristen Starnes-Ott

**Affiliations:** Department of Acute & Continuing Care, UTHealth School of Nursing, Houston, Texas

## Abstract

Do you have a topic in mind for a manuscript but can’t find the time or motivation to write it? This article provides step-by-step directions to encourage you to prioritize and plan your writing schedule.

Numerous topics encountered in both academic and clinical environments often have potential for publication; there are aspects of one’s specific practice or educational approach that can, and should, be shared with peers. Because the writing process is frequently perceived as a tedious, solitary, and time-consuming task, which, once in the form of a manuscript has the potential for rejection, it is all too easy to abandon the writing-for-publication process even before beginning (Happell, 2012). Practitioners in both academic and clinical practice settings may benefit from the following recommendations on establishing an individual writing practice.

## WRITING APPROACHES

Across all disciplines, the literature to facilitate a writing practice encourages would-be authors to view and prioritize their writing efforts as a routinely scheduled occurrence (Boice, 1989, 1990, 2000; Gray, 2010; Reeves, 2002; Zinsser, 2016). Boice (1989) and Gray (2010) provide substantial evidence to incentivize the concept of a scheduled writing approach by describing a research comparison of two groups of academic writers: one whose writing approach occurred in binges to meet specific deadlines vs. the other that spent 15 to 20 minutes a day putting pen to paper. The group that wrote daily with periodic encouragement and oversight penned or edited nine times more manuscript pages per year (N = 157 pages) than the group practicing "binge" writing or editing with colleagues either for a target deadline or when they perceived themselves as ready to write (N = 17 pages; Boice, 1989; Gray, 2010).

## MOTIVATIONAL SUGGESTIONS

For an individual working to establish authorship, recommendations to facilitate a writing practice are consistent. The first step is to schedule writing time on your planner, using the same approach as when you are scheduling departmental meetings, teaching a class, or managing clinical responsibilities. The schedule can be a simple, designated interval of 15 minutes carved out daily or a schedule such as every Monday, Wednesday, and Friday for 30 to 45 minutes. Exercising a dedicated writing schedule limits the tendency to procrastinate until facing an impending deadline and eliminates the "warm-up" period.

Once you have calendared writing appointments, consider the following suggestions for maintaining motivation:

1. Establish your writing appointment in the morning or during the time frame you designate as your most productive in the day. Mornings often work best, because, like exercise, it may be easier to begin writing when you are less likely to procrastinate or be distracted by daily disruptions and unexpected demands on your time. An established practice will also help minimize writing anxiety and "warm-up" time (Boice, 1990).
2. Have a designated writing space that is comfortable and pleasant to facilitate thinking and writing with few distractions (Boice, 1990).
3. Break your writing work into blocks or topic "chunks," such as an outline or the introduction of a piece you have been contemplating. A stepwise approach will make producing a full-length manuscript less daunting (Boice, 1990).
4. Start small. A case study, a clinical practice matter, or a topic of interest relevant to education or specialty practice are good starting points. The gradual evolution of your writing practice into larger undertakings, such as a review or research piece, will be less overwhelming when you’ve become comfortable with a routine practice of writing.
5. Once a topic has been identified, determine your audience and a target journal for publication. Preemptively identifying a suitable journal for your intended publication will serve to guide and direct how best to organize and present the information (O’Halloran & Doody, 2014).
6. As you develop the piece you are writing, use key sentences to create a preliminary "after-the-writing" outline. This exercise will help focus your efforts toward the central points you want to communicate (Gray, 2010).
7. Write the background or introduction while completing enrollments or other research processes, so when the statistical analyses or other results are complete, you will have already started, and perhaps even revised, a significant portion of the manuscript (Boice, 1990).
8. Allow initial drafts to be reviewed by a friend or professional colleague who is not an expert on your topic. Ask that person to assess whether the primary point is clear, because early in the writing process, your intent should be to focus on organization and flow of content (Boice, 1990).
9. Final-stage drafts should be reviewed by a colleague with technical content expertise who can diagnose areas of concern in the manuscript.
10. Consider working on two to three pieces at once rather than focusing on one. This practice may stimulate new thoughts and ideas as well as decrease the likelihood of tedium associated with working on only one project over an extended period (Boice, 1990).
11. Hold yourself accountable to your established schedule. Boice (1990) offers a unique, innovative strategy to keep you on task: You can prewrite a $25 check to a service group or organization you despise. If you fail to follow your writing schedule for a week, mail off your donation. Your scorn will keep you more on task than if you target an organization you admire or already support.
12. Seek out collaborators, especially for writing or completing a task you’ve had difficulty completing (Boice, 1990; Rosenthal, 2003). The value of meeting regularly and working together to maintain momentum may provide the impetus needed to complete a manuscript, particularly for a novice writer (Rosenthal, 2003).
13. Read and revisit literature on the practice of writing. Frequent recommendations for novice writers include *On Writing Well: The Classic Guide to Writing Nonfiction* (Zinsser, 2016) and *How to Write a Lot* (Silvia, 2007).

## DEVELOPING A PERSONAL WRITING PRACTICE

The effectiveness of an unfluctuating regular writing practice cannot be overemphasized. In our current academic and clinical environments, productivity and ideas for exploration increase substantially when potential authors commit to writing on a consistent basis, even for brief, scheduled periods. Regularly scheduled writing also enhances the starting process. An idea jotted down today may be interwoven with later ideas, which may stimulate entirely new areas of interest in research and practice.

In our experience, a personal writing practice is promoted by synchronous participation in a writing group. Although writing is a solitary endeavor, participation in small author groups that vary based on topic interest and development often augments an individual writing practice. For example, a doctor of nursing practice in our practice identified value in emphasizing the recent guideline changes for HPV vaccinations. Three providers from varied backgrounds met to discuss the focus of information and target the audience for the publication. Subsequently, each provider/author individually produced subtopic manuscripts, meeting again later to discuss organization and content within self-identified deadlines. The lead author provided early editorial flow of the first and second drafts. Utilizing the services of an onsite editor at the school of nursing helped actualize a final draft for submission. The group effort helped to keep each author on track while providing opportunities to discuss manuscript development and individual author obstacles.

## CLOSING THOUGHTS

As we have highlighted, writing is a process that requires dedication to acquire skills. Successful writers in both academic and nonacademic environments agree that productive writing requires a specific and well-developed procedure. The work of writing can be summed into the "3 Ps": (1) planning; (2) practice; and (3) persistence. These three attributes can feel overwhelming to a novice, or even to experienced writers who have numerous competing teaching, service, and clinical demands on their time. One approach to managing time constraints is through teamwork.

Although there are several approaches to writing one can use to establish a writing process, augmenting an individual writing practice with team writing and collaboration has proven most helpful. Taking the time to seek out potential writing collaborators or partners is often rewarding and results in long-term productivity for all members. Team science has been promoted by many funding agencies, academic units, and clinical settings and seamlessly integrates the team approach, as evidenced by the sharing of best practice guidelines, quality improvement project cycles, or research study results across disciplines.

The information presented here provides both steps to developing a writing practice and a real-time exemplar of melding this process with the support of a writing group. Establishing a writing practice using these suggestions, along with using the "3 Ps," can provide novice and experienced writers alike the keys to success.
